# Integrated Analysis Reveals Prognostic Value and Immune Correlates of CD86 Expression in Lower Grade Glioma

**DOI:** 10.3389/fonc.2021.654350

**Published:** 2021-04-19

**Authors:** Huaide Qiu, Wei Tian, Yikang He, Jiahui Li, Chuan He, Yongqiang Li, Ning Liu, Jianan Li

**Affiliations:** ^1^ Department of Rehabilitation Medicine, Jiangsu Shengze Hospital Affiliated to Nanjing Medical University, Suzhou, China; ^2^ Center of Rehabilitation Medicine, The First Affiliated Hospital of Nanjing Medical University, Nanjing, China; ^3^ Department of Neurosurgery, The First Affiliated Hospital of Nanjing Medical University, Nanjing, China; ^4^ Department of Rehabilitation Medicine, Zhongda Hospital, School of Medicine, Southeast University, Nanjing, China

**Keywords:** pan-cancer analysis, CD86, immune microenvironment, lower-grade glioma, prognosis

## Abstract

**Background:**

CD86 has great potential to be a new target of immunotherapy by regulating cancer immune response. However, it remains unclear whether CD86 is a friend or foe in lower-grade glioma (LGG).

**Methods:**

The prognostic value of CD86 expression in pan-cancer was analyzed using Cox regression and Kaplan-Meier analysis with data from the cancer genome atlas (TCGA). Cancer types where CD86 showed prognostic value in overall survival and disease-specific survival were identified for further analyses. The Chinese Glioma Genome Atlas (CGGA) dataset were utilized for external validation. Quantitative real-time PCR (qRT-PCR), Western blot (WB), and Immunohistochemistry (IHC) were conducted for further validation using surgical samples from Jiangsu Province hospital. The correlations between CD86 expression and tumor immunity were analyzed using the Estimation of Stromal and Immune cells in Malignant Tumours using Expression data (ESTIMATE) algorithm, Tumor IMmune Estimation Resource (TIMER) database, and expressions of immune checkpoint molecules. Gene Set Enrichment Analysis (GSEA) was performed using *clusterprofiler* r package to reveal potential pathways.

**Results:**

Pan-cancer survival analysis established CD86 expression as an unfavorable prognostic factor in tumor progression and survival for LGG. CD86 expression between Grade-II and Grade-III LGG was validated using qRT-PCR and WB. Additionally, CD86 expression in LGG with unmethylated O(6)-methylguanine-DNA-methyltransferase (MGMT) promoter was significantly higher than those with methylated MGMT (P<0.05), while in LGG with codeletion of 1p/19q it was significantly downregulated as opposed to those with non-codeletion (P<2.2*10-16). IHC staining validated that CD86 expression was correlated with MGMT status and X1p/19q subtypes, which was independent of tumor grade. Multivariate regression validated that CD86 expression acts as an unfavorable prognostic factor independent of clinicopathological factors in overall survival of LGG patients. Analysis of tumor immunity and GSEA revealed pivotal role of CD86 in immune response for LGG.

**Conclusions:**

Integrated analysis shows that CD86 is an unfavorable prognostic biomarker in LGG patients. Targeting CD86 may become a novel approach for immunotherapy of LGG.

## Introduction

Cancer cells escape surveillance of human immune system partly by activating immune checkpoint pathways, which leads to suppressed anti-cancer immune responses of the host (1, 2). To reactivate immune response against cancers, immune checkpoint inhibitors (ICIs) were developed and rose to be a revolution for cancer treatment (3). ICIs reinvigorate anti-cancer response by reactivating immune cells, and as a result enable clearance of cancer cells (4, 5). But well-established ICIs, including blockades targeting CTLA-4 and PD-1/PDL-1, only apply to a subset of cancer patients due to heterogeneous gene expressions and microenvironment across various cancer types (6), and as such novel therapeutic targets need to be considered (7, 8).

CD86 (B7–2), an immunoglobulin-like protein on antigen presenting cells (APCs), works in parallel with the CD80 (B7–1) as a natural ligand for CD28 and CTLA‐4 (9). CD86 promotes T-cell proliferation, function and survival by interacting with CD28 as a co-stimulator, while in activated T cells it interacts with CTLA-4 and acts as a suppressor (10, 11). In this bidirectional way, the interplay of CD86 with CD28 and CTLA‐4 are of great importance for immune responses against autoimmunity (12) and cancers (13). Notably, CD86 has shown higher affinity for binding to CTLA‐4 than that to CD28 (14), indicating the significance of CD86 in immunotherapeutic strategies based on CTLA-4 blockades, which have shown promising effects in treating solid tumors like melanoma (15) and mesothelioma (16) in clinical trials. Besides, CD86 expression was observed to be associated with unfavorable prognosis in myeloma (17) and leukemia (18). Due to the fact that CD86 may serve as a key regulator in cancer immune response *via* T-cell-mediated mechanisms, it has great potential to be a new target of immunotherapy. However, it remains unclear whether CD86 is a friend or foe in pan-cancer given its dual-edge role in regulating immune response.

In this study, we comprehensively analyzed the prognostic value of CD86 expression in pan-cancer, and found that CD86 acts as an unfavorable factor in the progression and prognosis of lower-grade glioma (LGG). External validation was conducted using surgical samples in our hospital and data from the Chinese Glioma Genome Atlas (CGGA) dataset. To predict survival probability of individual patient with CD86 expression and clinical features, a nomogram was developed and validated in both the cancer genome atlas (TCGA) and the CGGA datasets. Further, we explored the correlations between CD86 expression and tumor immunity of LGG samples, and Gene Set Enrichment Analysis (GSEA) was performed to reveal potential pathways.

## Materials and Methods

### Acquisition of Data and Ethics Approval

Normalized RNA Sequencing data with Fragments Per Kilobase of transcript per Million mapped reads (FPKM) in 33 different cancer types were downloaded from UCSC Xena (https://xena.ucsc.edu/), while clinical information was accessed using TCGAbiolinks R package on July 1^st^, 2020. Data for the validation cohort was accessed from the CGGA database (http://www.cgga.org.cn/), which was updated on June 14, 2020. Experimental validation was conducted using surgical samples from department of neurosurgery, the first affiliated hospital of Nanjing Medical University, also known as Jiangsu Province people’s hospital (JSPH). The web-lab validation was approved by the Institutional Review Board and the Ethics Committee of JSPH (No: 2020-SRFA-167), and all patients provided informed consent.

### Statistical Analysis

Survival analysis was performed using Cox regression analysis and Kaplan-Meier method, where Cox P-values and log-rank P-values were calculated. Between-group comparisons were conducted using Wilcoxon test (comparison between 2 groups) or Kruskal-Wallis test (comparison among 3 or more groups) (19). Spearman correlation was applied to determine significant correlations. Data were analyzed and visualized using R software 3.6.2, and P-value<0.05 was considered as statistically significant.

### Survival Analysis of CD86 Expression in Pan-Cancer

Survival analysis was conducted to estimate the prognostic value of CD86 expression on overall survival (OS) and disease-specific survival (DSS) in pan-cancer. In Cox regression analysis, Cox P-values and hazard ratios (HRs) with 95% confidence intervals (CI) were calculated; whereas, log-rank P-values and HRs with 95%CI were calculated in Kaplan-Meier method. Cancer types where CD86 expression showed prognostic value in OS and DSS were identified for further analyses.

### Correlations Between CD86 Expression and Tumor Progression

In the identified cancer types, the correlations between CD86 expression and tumor grade or stage were analyzed to explore the role of CD86 in tumor progression. The comparison of CD86 expression levels among different tumor stages/grades were explored. To investigate whether CD86 expression has independent prognostic value in overall survival, multivariate Cox regression was conducted to adjust the effect of demographic variables and tumor grade/stage. Exploration of cancer types for which CD86 expression showed prognostic value in tumor progression as well as in OS lead to the identification of LGG. CD86 expression profiles among different histological and molecular subtypes stratified by tumor grade of LGG were investigated.

### MRNA Extraction and qRT-PCR in JSPH LGG Samples

To further validate the results, 24 surgical samples of LGG (12 grade-II and 12 grade-III) were collected from JSPH and stored in liquid nitrogen. Total RNA was isolated from LGG samples using TRIzol reagent (Invitrogen, USA) according to the manufacturer’s instructions. Subsequently, quantitative real-time PCR (qRT-PCR) was employed to detect the expression levels of CD86 mRNA (forward: 5’-CTTTGCTTCTCTGCTGCTGT-3’ and reverse: 5’-GGCCATCACAAAGAGAATGTTAC-3’) with an ABI StepOnePlus system (Applied Biosystems) and TaqMan-based qRT-PCR assays. The primers for CD86 mRNA PCR were purchased from Guangzhou RiboBio (Guangzhou, China). β-Actin mRNA (forward: 5’-CACCCGCGAGTACAACCTTC-3’ and reverse: 5’-CCCATACCCACCATCACACC-3’) levels were measured for normalization. Data were analyzed using the 2^-ΔΔCt^ method with each test performed in triplicate.

### Immunohistochemical Analysis

The tissues for immunohistochemical analysis were fixed by formalin and embedded in paraffin. After being dewaxed in xylene and antigen retrieval, slides were incubated with Anti-CD86 antibody (ab243887, 1:200, Abcam, USA) overnight at 4°C, and then incubated with a Goat Anti-Rabbit IgG H&L antibody (1:50, Beyotime, China) at room temperature for 1 h, followed by incubation with ABC-peroxidase reagent for 1h, washed with PBS, stained with 3, 3-diaminobenzidine (30 mg dissolved in 100 mL Tris-buffer containing 0.03% H_2_O_2_) for 5 min, and rinsed in water before counterstained with hematoxylin. Each stained slide was individually reviewed and scored by two independent neuropathologists. Negative controls without primary antibody were included in all experiments to ensure the quality of the staining.

### Western Blot (WB) Analysis

Total protein was extracted from tissues using RIPA buffer (KenGEN, China), where protein concentrations were quantified with a BCA Protein Assay Kit (KenGEN, China). Protein was subjected to 10% SDS-PAGE and transferred to PVDF membranes (Millipore, USA). After being blocked with 5% non-fat milk for 2 h, the membranes were incubated overnight at 4°C with primary antibodies against CD86 (ab243887, 1:1000, Abcam, USA), followed by incubation with an HRP-conjugated secondary antibody (1: 3000, YIFEIXUE BIO TECH, China). β-Actin was used as the control (1:1,000, Beyotime, China).

### Validation of Prognostic Value of CD86 Expression in CGGA

The prognostic value of CD86 expression in the identified cancer was then validated in the CGGA LGG cohort (n=420). The Kaplan-Meier method was conducted to evaluate the prognostic value of CD86, which was further examined using univariate and multivariate Cox regression. Demographic information (age and gender), cancer type (primary/recurrent), tumor grade, and CD86 expression were incorporated in the regression analyses. If P values were unanimously less than 0.05 in both univariate and multivariate regressions, then CD86 expression was considered as an independent prognostic factor in overall survival of LGG.

### Development and Validation of a Nomogram

Using TCGA dataset, CD86 expression and clinical information, including gender, age, tumor grade, cancer type (primary or recurrent), chemotherapy (Yes or No), radiotherapy (Yes or No), and molecular subtypes was employed in univariate and multivariate Cox regressions to identify independent prognostic factors. Subsequently, a nomogram with independent prognostic factors was formulated and validated using the receiver operator characteristic (ROC) analysis and calibration at multiple time-points (20). Validation the nomogram was carried out in both TCGA and the CGGA datasets. Area under curves (AUCs) were calculated to evaluate the discrimination of the nomogram with AUC>0.7 being acceptable and AUC>0.8 being excellent (21, 22). Calibration was performed to compare the predicted probability and the actual observation, indicating the predicative accuracy of the nomogram.

### Exploration of CD86-Related Tumor Immunity and Gene Set Enrichment Analysis

Correlation analyses were conducted between CD86 expression and tumor immunity evaluated by tumor purity, immune cells, and immune checkpoint molecules to explore the potential mechanisms whereby CD86 affects prognosis. Tumor purity was measured by stromal score (SS) and immune score (IS), as calculated with the Estimation of Stromal and Immune cells in Malignant Tumours using Expression data (ESTIMATE) algorithm (23). The relationships between CD86 expression and immune cells were analyzed using Tumor IMmune Estimation Resource (TIMER) database (https://cistrome.shinyapps.io/timer/), an online web server that extracted data from gene expression profiles and calculated the abundance of tumor-infiltrating immune cells (24, 25), which was correlated to CD86 expression level with the purity-corrected partial Spearman method (25). Additionally, the association between CD86 expression and immune checkpoint molecules were delineated using Spearman correlation analysis. Correlation coefficients >0.7 were considered as strong correlation, while those falls in the range from 0.4 to 0.7 were interpretated as moderate correlation and values less than 0.4 as weak correlation (26). GSEA was performed using *clusterProfiler* r package (27) to identify the enriched terms in Gene Ontology (GO) and Kyoto Encyclopedia of Genes and Genomes (KEGG).

## Results

### Pan-Cancer Survival Analysis of CD86 Expression Identified Three Cancer Types

**Figure 1:**
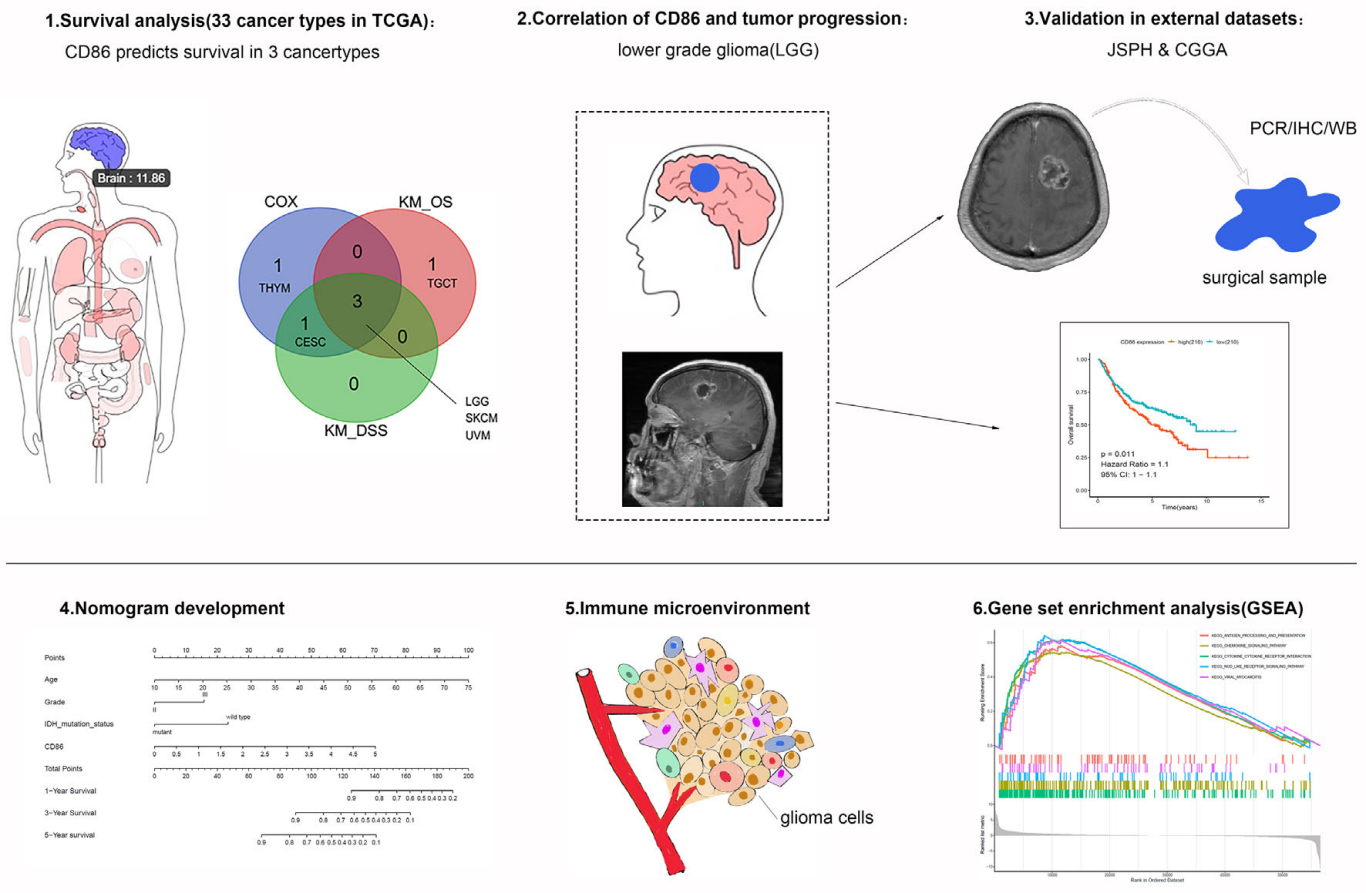
Schematic flowchart of the study process. The top left panel indicates that CD86 expression level of the brain is 11.86 [expression= Log_2_(TPM + 1)].

**Figure 2:**
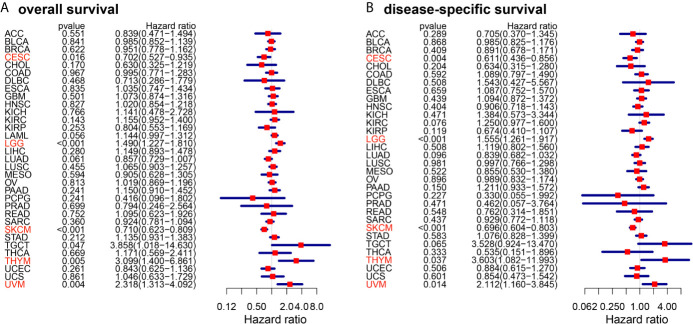
Forest plots of cox regression analysis with CD86 expressions in different cancer types. **(A)** Overall survival (OS). **(B)** Disease-specific survival (DSS). Cancer types with statistically significant prognostic value of CD86 in both OS and DSS are highlighted in red.

The schematic workflow of the study is presented in **Figure 1**, where the body image was downloaded from Gene Expression Profiling Interactive Analysis (GEPIA) (http://gepia.cancer-pku.cn/) (28). Survival Analysis of CD86 expression in pan-cancer was conducted to identify relevant cancer types. In Cox regression analysis, the results revealed that CD86 expression was significantly associated with survival rates in five cancer types, i.e., cervical squamous cell carcinoma and endocervical adenocarcinoma (CESC), LGG, skin cutaneous melanoma (SKCM), thymoma (THYM) and uveal melanoma (UVM) (**Figure 2**). Survival analysis on OS showed protective effects of CD86 expression in CESC (HR = 0.702, 95%CI [0.527, 0.935], Cox P = 0.016) and SKCM (HR= 0.710, 95%CI [0.623, 0.809], Cox P < 0.001), while unfavorable effects were demonstrated in LGG (HR= 1.490, 95%CI [1.227,1.810], Cox P < 0.001), THYM (HR = 3.099, 95%CI [1.400, 6.861], Cox P = 0.005) and UVM (HR = 2.318, 95%CI [1.313, 4.092], Cox P = 0.004) (**Figure 2A**). The results on DSS were in line with the OS analysis, showing similar effect of CD86 expression in the five cancer types: CESC (HR = 0.611, 95%CI [0.436, 0.856], Cox P = 0.004), LGG (HR = 1.555, 95%CI [1.261, 1.917], Cox P < 0.001), SKCM (HR = 0.696, 95%CI [0.604, 0.803], Cox P < 0.001), THYM (HR = 3.603, 95%CI [1.082, 11.993], Cox P = 0.037) and UVM (HR = 2.112, 95%CI [1.160, 3.845], Cox P = 0.014) (**Figure 2B**).

**Figure 3:**
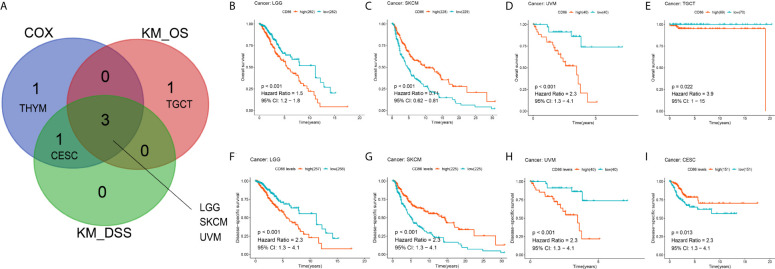
Kaplan-Meier analysis with CD86 expressions in different cancer types. **(A)** The Venn diagram of the identified cancer types in cox regression analysis and Kaplan-Meier method. **(B–E)** Kaplan-Meier survival curve showing the prognostic value of CD86 on OS in LGG **(B)**, SKCM **(C)**, UVM **(D)**, TGCT **(E)**. **(F–I)** Kaplan-Meier survival curve showing the prognostic value of CD86 on DSS in LGG **(F)**, SKCM **(G)**, UVM **(H)**, CESC **(I)**.

Using Kaplan-Meier method, we also conducted pan-cancer survival analysis of CD86 expression. CD86 was observed to be prognostic in four cancer types (**Figure 3A**), i.e., LGG (HR = 1.5, 95%CI [1.2, 1.8], log-rank P < 0.001) (**Figure 3B**), SKCM (HR = 0.71, 95%CI [0.62, 0.81], log-rank P < 0.001) (**Figure 3C**), UVM (HR = 2.3, 95%CI [1.3, 4.1], log-rank P < 0.001) (**Figure 3D**) and Testicular Germ Cell Tumor (TGCT; HR = 3.9, 95%CI [1, 15], log-rank P = 0.022) (**Figure 3E**). Similarly, CD86 expression demonstrated to be prognostic on DSS in four cancer types: LGG (HR = 2.3, 95%CI [1.3, 4.1], log-rank P < 0.001) (**Figure 3F**), SKCM (HR = 2.3, 95%CI [1.3, 4.1], log-rank P < 0.001) (**Figure 3G**), UVM (HR = 2.3, 95%CI [1.3, 4.1], log-rank P < 0.001) (**Figure 3H**), and CESC (HR = 2.3, 95%CI [1.3, 4.1], log-rank P = 0.013) (**Figure 3I**). The intersection of survival analysis with OS and DSS highlighted three cancer types (LGG, SKCM, and UVM), which indicated that CD86 expression has prognostic value in these three cancer types.

### CD86 Expression Was Correlated With Tumor Progression and Worse OS in LGG

**Figure 4:**
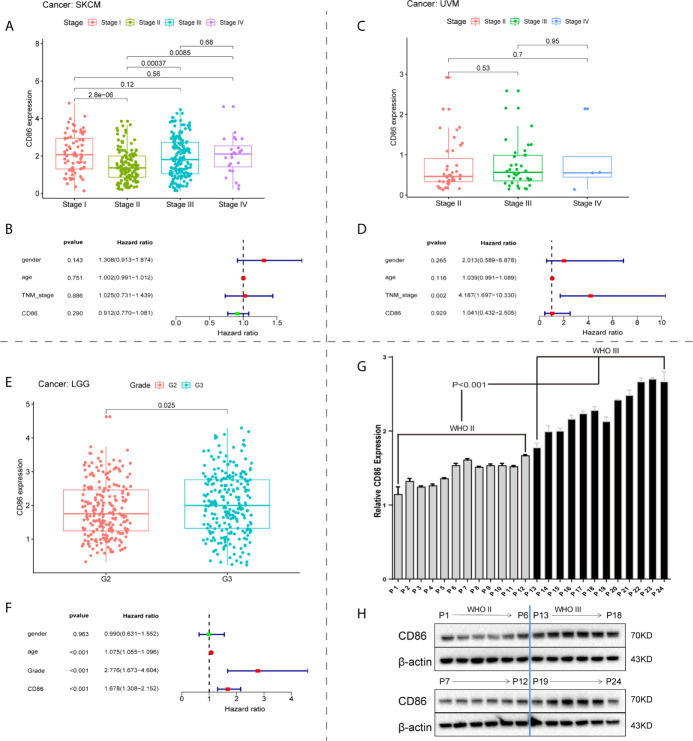
Correlations between CD86 expression and tumor progression. CD86 expression in different stages of SKCM **(A)** and UVM **(C)**. Multivariate regression analysis of CD86 expression, age, gender, and tumor stage for OS in SKCM **(B)** and UVM **(D)**. **(E)** CD86 expression between different grades of LGG. **(F)** Multivariate regression analysis of CD86 expression, age, gender, and tumor grade for OS in LGG. **(G)** CD86 mRNA expression evaluated by qRT-PCR in different grades of LGG. **(H)** CD86 protein expression evaluated by WB in different grades of LGG.

We investigated the correlations between CD86 expression and tumor progression in the identified cancer types: SKCM, UVM and LGG. Although CD86 expression was significantly altered among different tumor stages in SKCM (**Figure 4A**), no independent prognostic value in OS was observed (**Figure 4B**). In contrast, there was no significant correlation between CD86 expression and tumor stage of UVM (**Figure 4C**), neither was independent prognostic value of CD86 for UVM (**Figure 4D**). Higher CD86 expression was present in Grade-III LGG as compared to Grade-II (p=0.025), indicating a carcinogenetic effect of CD86 in LGG (**Figure 4E**). The multivariate regression analysis showed an independent prognostic value of CD86 in LGG on OS (HR = 1.678, 95%CI [1.308, 2.152], Cox P < 0.001) after variables including age, gender, and tumor grade were adjusted (**Figure 4F**). Consistent with bioinformatic analysis, *in vitro* experiments with 24 surgical samples of LGG using qRT-PCR (**Figure 4G**) and WB analysis (**Figure 4H**) indicated that CD86 expression in Grade-III LGG was significantly higher than that in Grade-II. Thus, CD86 was observed to be an unfavorable prognostic factor in tumor progression, OS, and DSS for LGG.

### CD86 Expression Was Correlated With Histological and Molecular Subtypes of LGG

**Figure 5:**
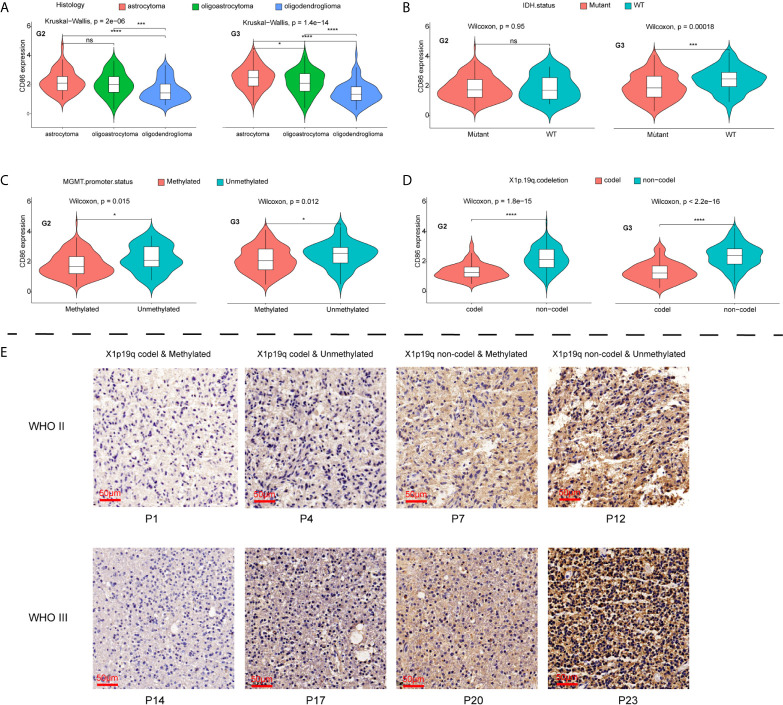
Comparisons of CD86 expression with different histological/molecular subtypes of LGG stratified by tumor grade. **(A)** CD86 expression in astrocytoma, oligoastrocytoma and oligodendroglioma. **(B)** CD86 expression in IDH mutant and WT of LGG. **(C)** CD86 expression in MGMT-methylated LGG versus unmethylated type. **(D)** CD86 expression in LGG with X1p/19q codeletion versus non-codeletion. **(E)** IHC staining of CD86 among different molecular subtypes regarding status on MGMT methylation and X1p/19q codeletion. *p<0.05 **p<0.01, ***p<0.001, ****p<0.0001, ns: not significant.

CD86 expression profiles among histological and molecular subtypes stratified by tumor grade in LGG were examined. Significantly higher expression of CD86 was observed in Grade-III astrocytoma as compared with oligoastrocytoma and oligodendroglioma of the same grade, while oligodendroglioma presented lower CD86 expression as opposed to oligoastrocytoma (P<1.4*10^-14^) (**Figure 5A**). Grade-II glioma showed the same trend between histological types, with no statistical difference detected in CD86 expressions between astrocytoma and oligoastrocytoma (**Figure 5A**). Besides, CD86 expression in MGMT-unmethylated LGG (Grade-II & Grade-III) was significantly higher than those with methylated MGMT (P<0.05) (**Figure 5B**). As shown in **Figure 5C**, markedly higher CD86 expressions were demonstrated in Grade-III glioma with wild-type (WT) isocitrate dehydrogenase (IDH) compared with IDH mutant (P<0.001), while no between-group significance was observed in Grade-II glioma. CD86 in LGG with codeletion of 1p/19q was significantly downregulated as opposed to those with non-codeletion (P<2.2*10^-16^) (**Figure 5D**). Immunohistochemistry (IHC) staining validated that CD86 expression was correlated with MGMT status and X1p/19q subtypes (**Figure 5E**), which is independent of tumor grade. IHC staining for 24 cases with LGG can be accessed in **Supplementary File S1**.

### CD86 Was an Unfavorable Prognostic Factor in CGGA LGG Patients

**Figure 6:**
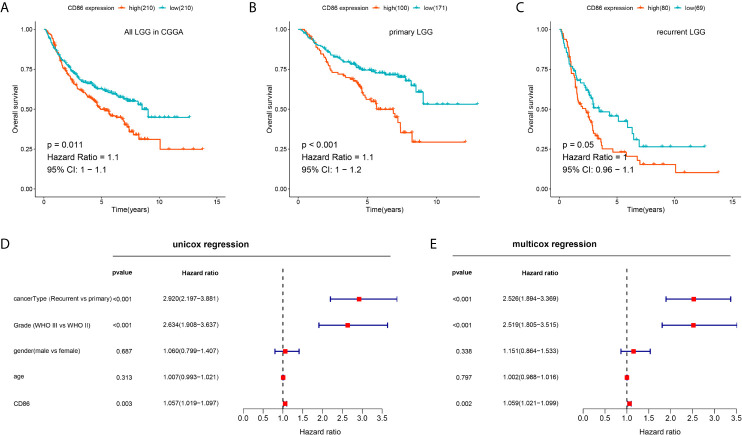
Validation of the prognostic value of CD86 for LGG in CGGA. **(A)** Kaplan-Meier analysis of CD86 expression and OS in all LGG. **(B)** Kaplan-Meier analysis of CD86 expression and OS in primary LGG. **(C)** Kaplan-Meier analysis of CD86 expression and OS in recurrent LGG. **(D)** Univariate Cox regression of CD86 expression, LGG cancer type (primary or recurrent), grade, gender and age. **(E)** Multivariate Cox regression using the same variables.

The prognostic performance of CD86 expression in LGG was validated in CGGA to determine whether the prognostic value of CD86 was independent of datasets. Kaplan-Meier analysis showed that CD86 expression was significantly correlated with survival rates in LGG (HR = 1.1, 95%CI [1, 1.1], log-rank P = 0.011) (**Figure 6A**), primary LGG (HR = 1.1, 95%CI [1, 1.2], log-rank P < 0.001) (**Figure 6B**), and recurrent LGG (HR = 1, 95%CI [0.96, 1.1], log-rank P = 0.05) (**Figure 6C**). The results of univariate and multivariate regression validated that CD86 acts as an unfavorable prognostic factor independent of clinicodemographic factors in overall survival of LGG patients (**Figures 6D, E**).

### Development and Validation of a Nomogram

**Figure 7:**
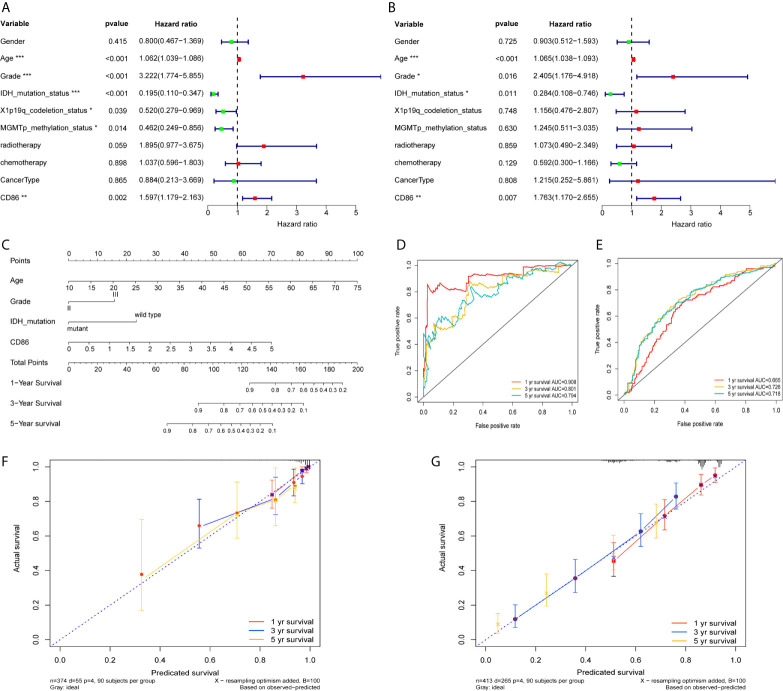
Development and validation of a Nomogram. Univariate Cox regression **(A)** and Multivariate Cox regression **(B)** with CD86 expression, demographic and clinicopathological factors; Red dots represent risk factor (HRs>1), while green dots represent protective factor (HRs<1). *P<0.05, **P<0.01, ***P<0.001. **(C)** Nomogram with independent prognostic factors. ROC curve analysis at 1 year, 3years, and 5 years using TCGA dataset **(D)** and the CGGA dataset **(E)**. Calibration plot at 1 year, 3years, and 5 years in TCGA **(F)** and the CGGA **(G)**.

Univariate Cox regression revealed prognostic values of CD86 expression, age, tumor grade, as well as molecular subtypes including IDH mutation status, X1p/19q codeletion, and MGMT methylation (**Figure 7A**); whereas, multivariate Cox regression showed independent prognostic roles of CD86 expression, age, tumor grade, and IDH mutation status in overall survival of LGG (**Figure 7B**). A nomogram with these independent factors was formulated to predict an individualized probability of survival (**Figure 7C**). The ROC curve analysis of the nomogram in TCGA dataset showed acceptable to excellent accuracy in classification with 1-year AUC of 0.904, 3-year AUC of 0.801, 5-year AUC of 0.794 (**Figure 7D**). Additionally, ROC analysis in the CGGA dataset validated the classification performance with 1-year AUC of 0.665, 3-year AUC of 0.726, 5-year AUC of 0.728 (**Figure 7E**). Moreover, calibration revealed adequate prediction accuracy of the nomogram at multiple timepoints in TCGA (**Figure 7F**) and CGGA (**Figure 7G**).

### CD86 Expression Was Correlated With Tumor Immunity and Implicated in Immune-Related Pathways

**Figure 8:**
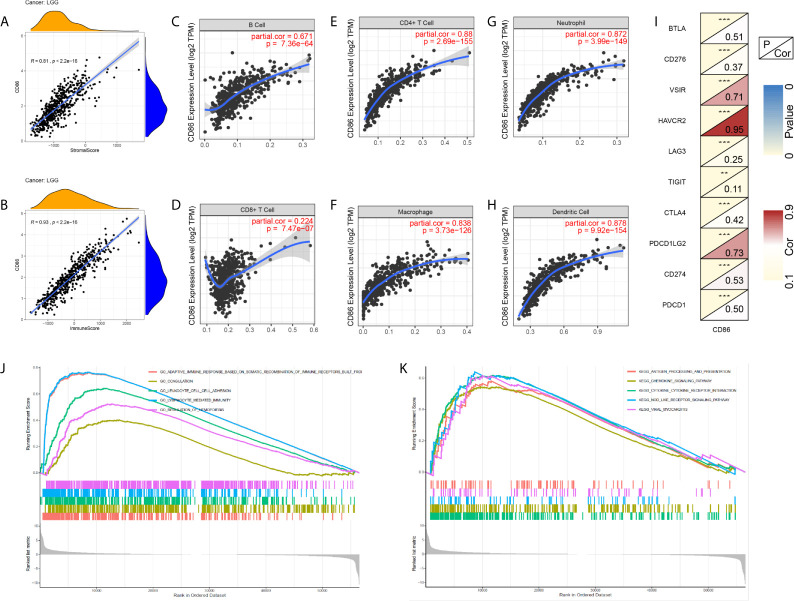
Exploration of CD86-related tumor immunity and GSEA. **(A)** Correlations between CD86 expression and Stromal Score. **(B)** Correlations between CD86 expression and Immune Score. Correlations between CD86 expression and different immune cells: B cell **(C)**, CD8 T cell **(D)**, CD4 T cell **(E)**, macrophage **(F)**, neutrophil **(G)**, and dendritic cell **(H)**. **(I)** Correlations between CD86 expression and different immune checkpoint molecules. **(J)** GSEA of GO terms. **(K)** GSEA in KEGG pathway. **p<0.01, ***p<0.001.

As shown in **Figures 8A**, **B**, SS and IS were both significantly correlated with CD86 expression (r>0.7, P<2.2*10^-16^), indicating CD86 could serve as a biomarker in tumor purity. Spearman correlation analysis demonstrated strong correlations of CD86 expression with CD4+ cells (**Figure 8E**), macrophage (**Figure 8F**), neutrophil (**Figure 8G**), as well as with dendritic cells (**Figure 8H**) using TIMER (r>0.7, P<0.0001). Moderate correlation was also observed between CD86 expression and B cells (**Figure 8C**), and there was weak correlation between CD86 expression and CD8+ cells (**Figure 8D**). Meanwhile, we found that CD86 expression correlated with multiple immune checkpoint molecules, including VSIR, HAVCR2, and PDCD1LG2 (PD-L2) (r>0.7, P<0.0001) (**Figure 8I**). Additionally, CD86 levels was associated with BTLA, CTLA4, CD274 (PD-L1), and PDCD1 (PD1) with moderate correlation (r>0.4, P<0.001) (**Figure 8I**).

Subsequently, GSEA was conducted to explore the underlying mechanisms whereby CD86 expression may alter prognosis in LGG. The results of GO analysis showed that CD86 was significantly enriched in adaptive immune response based on somatic recombination of immune receptors, coagulation, leukocyte cell-cell adhesion, and lymphocyte mediated immunity (**Figure 8J**). In KEGG analysis, CD86 was significantly enriched in antigen processing and presentation, chemokine signaling pathway, and cytokine-cytokine receptor interaction (**Figure 8K**).

## Discussion

In the present study, pan-cancer survival analyses revealed prognostic values of CD86 expression in three cancer types, i.e., LGG, SKCM and UVM. CD86 demonstrated to be an unfavorable factor independent of clinicodemographic variables in tumor progression and prognosis for LGG, which was validated by qRT-PCR and WB in LGG samples, as well as a real-world cohort in CGGA. Additionally, data from TCGA showed CD86 expression was associated with aggressive molecular subtypes of LGG, and IHC staining of surgical samples confirmed these associations. To predict an individualized probability of survival, a nomogram was developed with TCGA dataset, showing adequate classification performance and predictive accuracy in TCGA as well as the CGGA dataset. To explore potential mechanisms by which CD86 acts as an unfavorable prognostic factor in LGG, analysis of tumor immunity and GSEA revealed pivotal role of CD86 in immune response for LGG.

Although CD86 has been reported to be associated with poor prognosis in chronic lymphocytic leukemia (9), myeloma (29), and overall glioma (30), there was no report of its prognostic value in LGG and melanoma. As shown in the present study, CD86 expression level was significantly correlated with worse survival and it was upregulated as the tumor grade increases in LGG. Besides, univariate and multivariate Cox regression validated the independent prognostic value of CD86. Further, analysis of the correlations between CD86 expression and molecular subtypes of LGG indicated that CD86 expression was significantly higher in MGMT-unmethylated type and LGG with non-codeletion of 1p/19q. Low MGMT unmethylation has been established to be associated with poor survival of glioma according to previous studies (31–33), while IDH mutant with 1p/19q codeletion has been observed to have better therapeutic response and clinical outcomes compared to those with non-codeletion (34–36). Therefore, CD86 may alter the malignant processes of LGG by interacting with pathways related to MGMT status and 1p/19q codeletion, which could be relevant to treatment decisions for LGG patients.

Further, we formulated a nomogram to guide clinical practice in an individualized manner, and its predictive performance was validated across different datasets. Although many previous studies have adopted nomogram models in predicting overall survival of LGG patients, most of them (37–39) suffered from a lack of external validation. Our study, on the other hand, offered solid external validation with ROC analysis and calibration plot and the nomogram demonstrated to be clinically relevant, discriminant and accurate in predicting survival outcomes.

To further investigate on the mechanisms, the correlations between CD86 expression and immunity were comprehensively explored. The results indicated that CD86 expression was significantly associated with TME, which has been identified as a key factor in tumor progression and therapeutic response (40, 41). Specifically, we found strong correlations of CD86 expression with immune infiltration of CD4+ cells, macrophage, neutrophil and dendritic cells. These results were consistent with previous studies (38, 42) indicating higher levels of immune cell infiltration may contribute to worse prognosis of LGG. Additionally, CD86 levels demonstrated strong correlations with multiple immune checkpoint molecules, including VSIR, HAVCR2, and PDCD1LG2 (PD-L2). Although there was no report of VSIR and HAVCR2 in LGG, PD-L2 was observed to be an unfavorable prognosticator in tumor progression and prognosis for LGG patients (43). Likewise, CD86 could be a prognostic biomarker and serves as a potential therapeutic target for LGG patients.

To our best knowledge, this article presents the first report on the prognostic value of CD86 expression in pan-cancer. CD86 expression demonstrated to be an unfavorable prognostic factor in survival and tumor progression for LGG patients, thereby serving as potential target of immunotherapy. However, a cause-effect relationship of CD86 expression with prognosis could not be established in the present study. Further investigations about downstream mechanisms arewfi 2 needed, while potential pathways shown in GSEA suggested possible directions.

## Conclusion

In summary, CD86 expression is associated with tumor progression and prognosis for LGG patients, where its prognostic value was observed to be independent of clinical features. Besides, CD86 expression was correlated with levels of tumor-infiltrated immune cells and expressions of immune checkpoint molecules. CD86 could be a novel biomarker in the prognosis and treatment of LGG.

## Data Availability Statement

Publicly available datasets were analyzed in this study. This data can be found here: [https://xena.ucsc.edu/, http://www.cgga.org.cn/].

## Ethics Statement

The studies involving human participants were reviewed and approved by the Institutional Review Board and the Ethics Committee of JSPH (No: 2020-SRFA-167). The patients/participants provided their written informed consent to participate in this study.

## Author Contributions

HQ: Conceptualization, Data curation, Formal analysis, Roles/Writing - original draft, Writing - review & editing. WT: Lab investigation and Methodology. YH: Roles/Writing - original draft, Writing - review & editing. JHL, CH, and YL: Writing and revision of the draft. NL, and JNL: Funding acquisition, Methodology, Project administration, Resources, Supervision.

## Funding

This study was funded by The Introduced Project of Suzhou Clinical Medical Expert Team (SZYJTD201725), the Nanjing Municipal Science and Technology Bureau (No. 2019060002), the Postgraduate Research & Practice Innovation Program of Jiangsu Province(SJCX20_0478), and Key Project of Jiangsu Provincial Department of Science and Technology (BE2017007-5).

## Conflict of Interest

The authors declare that the research was conducted in the absence of any commercial or financial relationships that could be construed as a potential conflict of interest.

## Supplementary Material

Supplementary File S1 IHC staining of CD86 for 24 cases with LGG.
