# Supplementary figures and images for: Integrated Analysis Reveals Prognostic Value and Immune Correlates of CD86 Expression in Lower Grade Glioma
Source: Front Oncol. 2021 Apr 19;11:654350. doi: 10.3389/fonc.2021.654350 (PMC8089378; doi:10.3389/fonc.2021.654350)

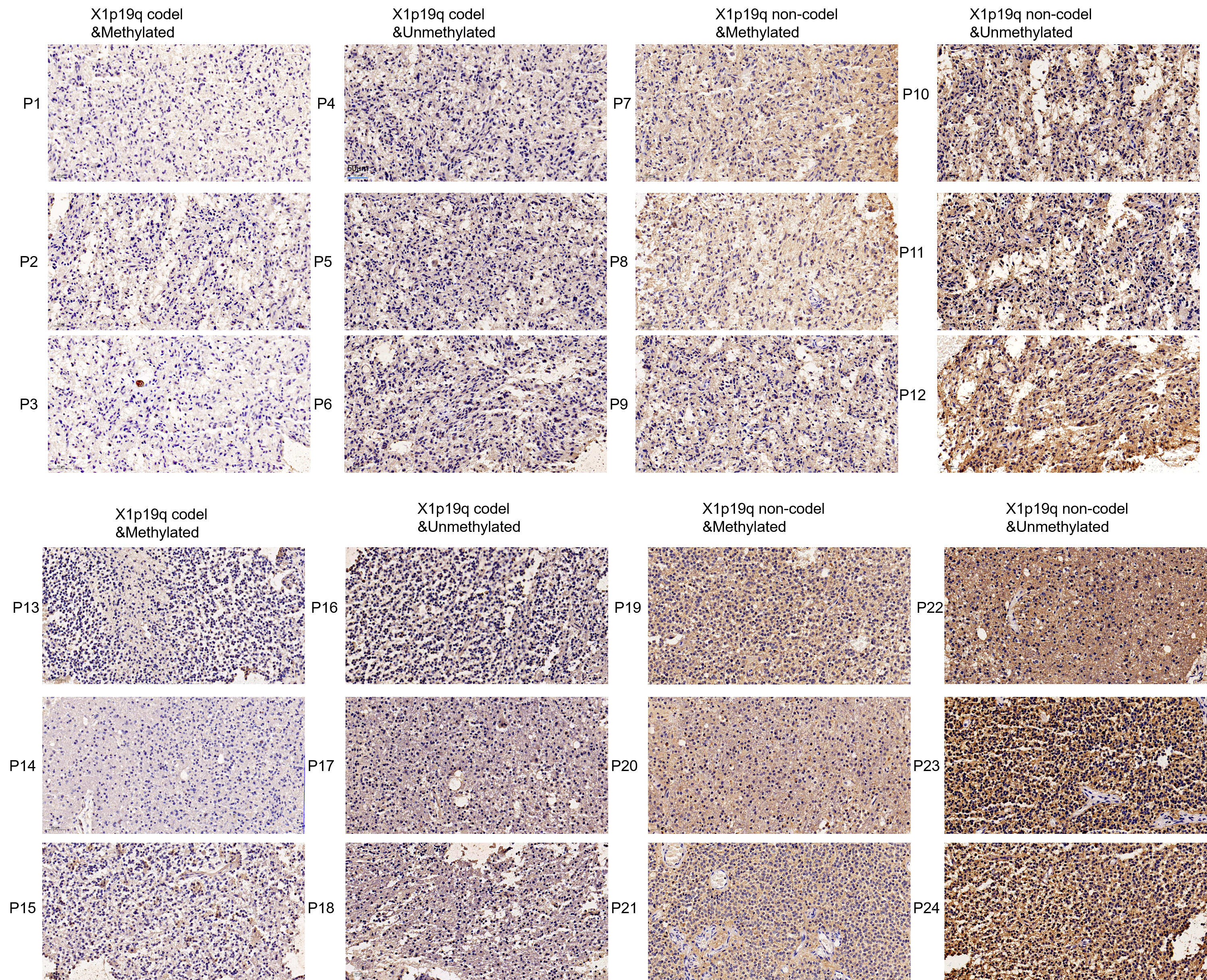

Supplement: Supplementary File S1 — IHC staining of CD86 for 24 cases with LGG. [file Image_1.tif]
